# Triglyceride-mediated influence of serum angiopoietin-like protein 8 on subclinical atherosclerosis in type 2 diabetic patients: results from the GDMD study in China

**DOI:** 10.1186/s12933-018-0687-y

**Published:** 2018-07-15

**Authors:** Tianpeng Zheng, Bo Ge, Hongbo Liu, Bo Chen, Linyuan Qin, Liuping Xiao, Jianfei Song

**Affiliations:** 1 Department of Endocrinology and Metabolism, The Second Affiliated Hospital of Guilin Medical University, 212 Renmin Road, Guilin, 541199 Guangxi People’s Republic of China; 2 Department of Urology, The Second Affiliated Hospital of Guilin Medical University, Guilin, Guangxi People’s Republic of China; 3 Department of Laboratory Medicine, The Second Affiliated Hospital of Guilin Medical University, Guilin, Guangxi People’s Republic of China; 4 Department of Human Anatomy, Southwest Medical University, Luzhou, Sichuan People’s Republic of China; 5 Department of Epidemiology and Health Statistics, Guilin Medical University, Guilin, Guangxi People’s Republic of China; 6 Department of Thoracic and Cardiovascular Surgery, The Second Affiliated Hospital of Guilin Medical University, Guilin, Guangxi People’s Republic of China

**Keywords:** ANGPTL8, Atherosclerosis, Type 2 diabetes, Triglyceride, Insulin resistance, Hyperglycemia

## Abstract

**Background:**

Hypertriglyceridemia, insulin resistance and hyperglycemia are risk factors for atherosclerosis in type 2 diabetes. Angiopoietin-like protein 8 (ANGPTL8) is a newly identified liver-derived hormone related to these risk factors. Hence, we aimed to explore the correlations between serum levels of ANGPTL8 and subclinical atherosclerosis in type 2 diabetes.

**Methods:**

We measured serum ANGPTL8, blood lipids, blood glucose, common carotid artery Intima-Media Thickness (c-IMT) and calculated homeostasis model assessment of insulin resistance in (1) control subjects (n = 100), (2) type 2 diabetic patients without subclinical atherosclerosis (n = 100), and (3) type 2 diabetic patients with subclinical atherosclerosis (n = 100).

**Results:**

Serum levels of ANGPTL8 and triglyceride (TG) were significantly increased in type 2 diabetic patients with subclinical atherosclerosis as compared with type 2 diabetic patients without subclinical atherosclerosis and control subjects (P < 0.001). ANGPTL8 was positively associated with age, TG, diabetes duration, and c-IMT in type 2 diabetes. Logistic regression analysis revealed that ANGPTL8 had higher odds of having subclinical atherosclerosis [odds ratio (OR) 2.90, 95% confidence interval (CI) 1.48–5.70, P = 0.002] in type 2 diabetes. Mediation analysis indicated that TG acted as a partial mediator in the relationship between ANGPTL8 and c-IMT.

**Conclusions:**

TG partially mediates the positive relationship between ANGPTL8 and c-IMT. Our data provide the first evidence for a strong link between ANGPTL8 and subclinical atherosclerosis, suggesting ANGPTL8 to be a new biomarker for subclinical atherosclerosis in type 2 diabetes.

## Background

Type 2 diabetes is a chronic multisystem disease associated with a cluster of vascular complications [1]. Atherosclerotic complications are the leading causes of morbidity and mortality among type 2 diabetic patients [2]. The detailed pathogenesis of atherosclerosis in type 2 diabetes is still not entirely clear, therefore, the identification of novel biomarkers and underlying mechanisms related to subclinical atherosclerosis in type 2 diabetes is of critical importance.

Angiopoietin-like protein 8 (ANGPTL8) (alternatively named refeeding-induced fat and liver [RIFL], lipasin, TD26) is a secreted protein of 198 amino acids primarily expressed in liver and adipose tissues. A meta-analysis study by Yue et al. revealed that type 2 diabetic patients had higher circulating levels of ANGPTL8 compared with those without type 2 diabetes, suggesting a relationship between increased ANGPTL8 and glucose metabolism [3]. In addition to its relationship with glucose metabolism, ANGPTL8 has also been implicated in both lipid metabolism and insulin resistance, all of which are thought to be involved in the pathogenesis of atherosclerosis [4–6]. Therefore, we were motivated to hypothesize that increased circulating levels of ANGPTL8 might be positively associated with common carotid artery Intima-Media Thickness (c-IMT) and serve as a novel biomarker for atherosclerosis in type 2 diabetes. To date, no study has ever evaluated the association between circulating levels of ANGPTL8 and c-IMT or considered the possibility of identifying ANGPTL8 as a risk biomarker for subclinical atherosclerosis in type 2 diabetes.

Consequently, the objective of the present study was to answer the three following questions: (1) Whether circulating levels of ANGPTL8 are increased in type 2 diabetic patients with subclinical atherosclerosis. (2) Is the increase in circulating levels of ANGPTL8 positively associated with subclinical atherosclerosis in type 2 diabetes? (3) If the second answer is yes, what factors mediate this positive relationship between ANGPTL8 and subclinical atherosclerosis in type 2 diabetes?

## Methods

### Study population

This was a cross-sectional analysis of baseline data from Guangxi Diabetes and Metabolic Disorders (GDMD) study aimed to investigate the etiology and comorbidities of type 2 diabetes and metabolic syndrome [7]. Participants were enrolled between February 2016 and May 2016 at the Medical Examination Center of the Affiliated Hospital of Guilin Medical University. A total of 300 Chinese subjects were enrolled, of which 100 control subjects with normal glucose tolerance (NGT), 100 type 2 diabetic patients with subclinical atherosclerosis and 100 type 2 diabetic patients without subclinical atherosclerosis. Exclusion criteria were as follows: (1) any evidence of acute diabetic complications, acute inflammatory diseases, malignancy, heart, liver, kidney, and respiratory failure. (2) Use of possible or known drugs affecting triglyceride (TG) for more than 3 months or at any time within 12 months before the recruitment. (3) Insulin use. (4) Subjects with incomplete data. The study was approved by the Ethics Committee of Affiliated Hospital of Guilin Medical University, and written informed consent has been obtained from each participant after full explanation of the purpose and nature of our study. This study was registered on the Chinese Clinical Trial Registry (ChiCTR-EPC-14005273).

### Measurements

A standard questionnaire was administered by trained staff to the patients to record demographic and clinical data. Anthropometric parameters were measured and calculated as previously described [8]. Participants were instructed to maintain their usual diet and physical activity for at least 3 days before the examination. After an overnight fast of 10 h or longer, venous blood samples were collected to measure fasting plasma glucose (FPG), fasting insulin, HbA1c, blood lipids and ANGPTL8, blood samples were also drawn at 120 min after a 75 g glucose load to measure glucose in controls subjects with normal glucose tolerance. Plasma glucose levels, insulin, TC, TG, HDL-C and LDL-C were measured as previously described [1]. Serum levels of ANGPTL8 were quantified using a commercially available ELISA kit (Wuhan Eiaab Science, Wuhan, China) according to the manufacturer’s instructions. The determination of c-IMT was performed as previously described [9]. The subclinical atherosclerosis was defined as c-IMT > 0.9 mm and/or presence of AS plaques in the carotid artery, the presence of carotid plaques was defined as focal echo structures encroaching into the arterial lumen of at least 0.5 mm or 50% of the surrounding c-IMT value, or when c-IMT was > 1.5 mm as measured from the media-adventitia interface to the intima-lumen interface [1]. The homeostasis model assessment of insulin resistance (HOMA-IR) was calculated as previously reported (HOMA-IR = Fasting glucose * Fasting insulin/22.5) [1].

### Statistical analysis

Statistical analyses were conducted using SPSS 16.0 software. The distribution of continuous variables were checked for normality before analysis, TG, fasting insulin and HOMA-IR were normalized by a logarithm transformation. Continuous variables were described as mean ± SD, categorical variables were described as frequencies and proportions. Clinical and biochemical parameters were compared by ANOVA, Chi square, or t test. Partial correlation analysis and multivariate regression analysis controlling for age, BMI and gender were estimated to examine the relationship between variables. A binary logistic regression analysis was used to estimate the association of age, TG, duration of diabetes and ANGPTL8 with the odds of subclinical atherosclerosis in type 2 diabetes.

To determine whether the relationship between ANGPTL8 and c-IMT was mediated by hypertriglyceridemia, insulin resistance and hyperglycemia, mediation analysis were conducted based on the procedures outlined by Baron and Kenny [10]. A three-step linear regression model was constructed as follows: (1) Y = cX + e1 (2) M = aX + e2 (3) Y = c’X + bM + e3, where X is the independent variable (ANGPTL8), Y is the dependent variable (c-IMT), M is the mediator, a is the regression coefficient for the association between ANGPTL8 and mediator, b is the regression coefficient for the association between mediator and c-IMT, c is the regression coefficient for the association between ANGPTL8 and c-IMT, and c’ is the effect of ANGPTL8 on c-IMT while controlling for the indirect effect. A Sobel Test was further performed to test mediation: First, the indirect effect was calculated by the effect of the independent variable on the mediator obtained from the first regression model multiplied by the effect of the mediator on the dependent variable from the third regression model. Second, the indirect effect was further divided by its standard error and a z test was performed [11]. An indirect ratio was used to present the strength of mediation: ([a*b]/c).

In this analysis, four conditions used to establish mediation were as follows: (1) the independent variable should be significantly associated with the dependent variable; (2) the independent variable should be significantly associated with the mediator; (3) the mediator should be significantly associated with the dependent variable; (4) the relationship between the dependent and independent variable should be attenuated when the mediator is included in the regression model.

## Results

### Clinical and laboratory characteristics

Table 1 summarized the clinical characteristics of the three groups including control subjects with normal glucose tolerance, type 2 diabetic patients without subclinical atherosclerosis, and type 2 diabetic patients with subclinical atherosclerosis. Our data indicated that statin use, TG, duration of diabetes, c-IMT and ANGPTL8 in type 2 diabetic patients with subclinical atherosclerosis had a significant increase than in control subjects with NGT and type 2 diabetic patients without subclinical atherosclerosis, whereas leisure-time physical activity had a significant decrease in type 2 diabetic patients with subclinical atherosclerosis than in control subjects. There were no significant differences in age, sex, BMI, current smoking, habitual alcohol drinking, SBP, DBP, TC, LDL-C and HDL-C among three groups.

**Table 1:** Clinical characteristics in control subjects with normal glucose tolerance (NGT), type 2 diabetes (T2DM) patients without atherosclerosis, and T2DM patients with atherosclerosis

Characteristics	Control subjects with NGT (n = 100)	T2DM without atherosclerosis (n = 100)	T2DM with atherosclerosis (n = 100)	P value
Age (years)	56.4 ± 7.6	56.2 ± 8.4	57.2 ± 7.7	0.628
Percent men (%)	54	56	49	0.593
Body mass index (kg/m^2^)	24.7 ± 3.7	24.4 ± 3.0	25.0 ± 3.6	0.400
Current smoking (%)	21	23	25	0.798
Habitual alcohol drinking (%)	26	22	28	0.611
Leisure-time physical activity (%)	67	52	49	0.023
Statin use (%)	14	32	42	< 0.001
NSAID use (%)	5	34	38	< 0.001
SBP^a^	132 ± 15	134 ± 18	134 ± 13	0.398
DBP^a^	72 ± 9	75 ± 11	74 ± 12	0.068
TG (mmol/L)^a^	1.31 (0.92,1.77)	1.62 (1.07,2.50)	2.04 (1.26,3.41)	< 0.001
TC (mmol/L)^a^	4.83 ± 0.90	5.10 ± 0.85	4.94 ± 1.09	0.088
LDL-C (mmol/L)^a^	2.88 ± 0.69	3.08 ± 1.06	3.16 ± 0.89	0.082
HDL-C (mmol/L)^a^	1.23 ± 0.33	1.16 ± 0.41	1.19 ± 0.33	0.306
Fasting plasma glucose (mmol/L)	4.83 ± 0.63	8.62 ± 1.70	8.09 ± 1.64	< 0.001
Fasting insulin (μU/mL)	6.32 (5.17,8.77)	8.43 (7.09,10.88)	7.83 (5.99,11.06)	< 0.001
HOMA-IR	1.41 (1.05, 1.93)	3.23 (2.45, 4.33)	2.92 (2.13, 3.73)	< 0.001
Duration of diabetes (years)	–	6.7 ± 3.1	7.7 ± 4.4	< 0.001
HbA1c (%)^a^	5.1 ± 0.4	7.8 ± 1.3	7.7 ± 1.0	< 0.001
HbA1c (mmol/mol)^a^	32.6 ± 4.1	62.0 ± 14.1	60.4 ± 10.7	< 0.001
c-IMT (mm)^a^	0.54 ± 0.08	0.74 ± 0.09	0.90 ± 0.14	< 0.001
ANGPTL8^a^ (pg/mL)	731 ± 285	1360 ± 310	1585 ± 555	< 0.001

Data were expressed as mean ± standard deviation, median (interquartile range), or percentage for continuous various and categorical variables, respectively. Cigarette smoking was defined as having smoked at least 100 cigarettes in one’s lifetime. Regular leisure-time physical activity was defined as participation in ≥ 30 min of moderate or vigorous activity per day at least 3 days per week

^a^Adjusted for age, gender, and BMI

### Correlation between ANGPTL8 and clinical parameters

**Fig. 1:**
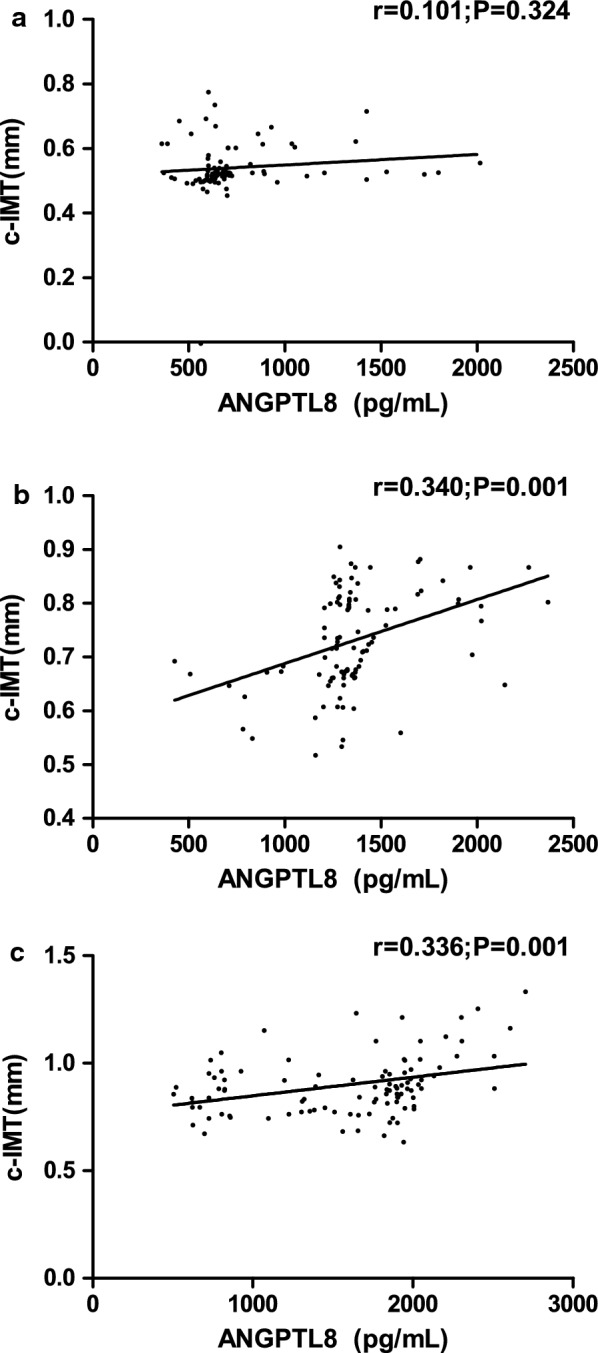
**a** Correlations between ANGPTL8 and c-IMT in control subjects with normal glucose tolerance (NGT). **b** Correlations between ANGPTL8 and c-IMT in T2DM patients without atherosclerosis. **c** Correlations between ANGPTL8 and c-IMT in T2DM patients with atherosclerosis

As shown by partial correlation analysis (Table 2 and Fig. 1), serum levels of ANGPTL8 were positively and significantly correlated with TG, duration of diabetes and c-IMT in type 2 diabetic patients with and without subclinical atherosclerosis groups (all P < 0.05) after adjustment for age, sex and BMI. No significant correlations were found between ANGPTL8 and SBP, DBP, TC, HDL-C, LDL-C, fasting plasma glucose, fasting insulin, HOMA-IR, HbA1c in all three groups. Multivariate regression analysis showed that TG, duration of diabetes and c-IMT were independently related factors influencing serum levels of ANGPTL8 in both type 2 diabetic patients with and without subclinical atherosclerosis groups (Table 3).

**Table 2:** Correlations analysis of variables associated with circulating ANGPTL8 levels in control subjects with normal glucose tolerance (NGT), T2DM patients without atherosclerosis and T2DM patients with atherosclerosis

	Control subjects with NGT		T2DM without atherosclerosis		T2DM with atherosclerosis	
	r	P	r	P	r	P
Age	0.188	0.061	0.330	0.001	0.206	0.039
BMI	0.000	0.997	− 0.029	0.778	0.235	0.018
SBP^a^	− 0.031	0.763	− 0.042	0.686	0.106	0.301
DBP^a^	0.126	0.218	0.033	0.746	− 0.037	0.723
TG^a^	0.141	0.169	0.379	< 0.001	0.406	< 0.001
TC^a^	− 0.055	0.596	0.020	0.848	0.083	0.419
LDL-C^a^	− 0.032	0.755	0.040	0.696	− 0.050	0.625
HDL-C^a^	− 0.144	0.158	0.088	0.392	0.075	0.466
Fasting plasma glucose^a^	0.127	0.216	0.087	0.395	− 0.009	0.932
Fasting insulin^a^	− 0.076	0.461	0.106	0.301	0.187	0.067
HOMA-IR^a^	− 0.028	0.789	0.114	0.265	0.160	0.117
HbA1c^a^	0.025	0.810	0.150	0.143	− 0.120	0.243
Duration of diabetes^a^	–	–	0.250	0.013	0.306	0.002
c-IMT (mm)^a^	0.101	0.324	0.340	0.001	0.336	0.001

^a^P value determined by partial correlation analysis (age, sex, and BMI adjusted)

**Table 3:** Multivariate regression analysis with ANGPTL8 as dependent variable

	Control subjects with NGT		T2DM without atherosclerosis^a^		T2DM with atherosclerosis^a^	
	β	P	β	P	β	P
SBP^a^	− 0.032	0.763	− 0.040	0.686	0.103	0.301
DBP^a^	0.132	0.218	0.033	0.746	− 0.035	0.723
TG^a^	0.142	0.169	0.364	< 0.001	0.400	< 0.001
TC^a^	− 0.055	0.596	0.019	0.848	0.080	0.419
LDL-C^a^	− 0.032	0.755	0.039	0.696	− 0.050	0.625
HDL-C^a^	− 0.142	0.158	0.083	0.392	0.072	0.466
Fasting plasma glucose^a^	0.125	0.216	0.083	0.395	− 0.008	0.932
Fasting insulin^a^	− 0.075	0.461	0.102	0.301	0.183	0.67
HOMA-IR^a^	− 0.027	0.789	0.109	0.265	0.158	0.117
HbA1c^a^	0.025	0.810	0.141	0.143	− 0.117	0.243
Duration of diabetes^a^	–	–	0.242	0.013	0.294	0.002
c-IMT (mm)^a^	0.100	0.324	0.333	0.001	0.322	0.001

^a^Age, sex, and BMI adjusted

### Mediation analysis

**Fig. 2:**
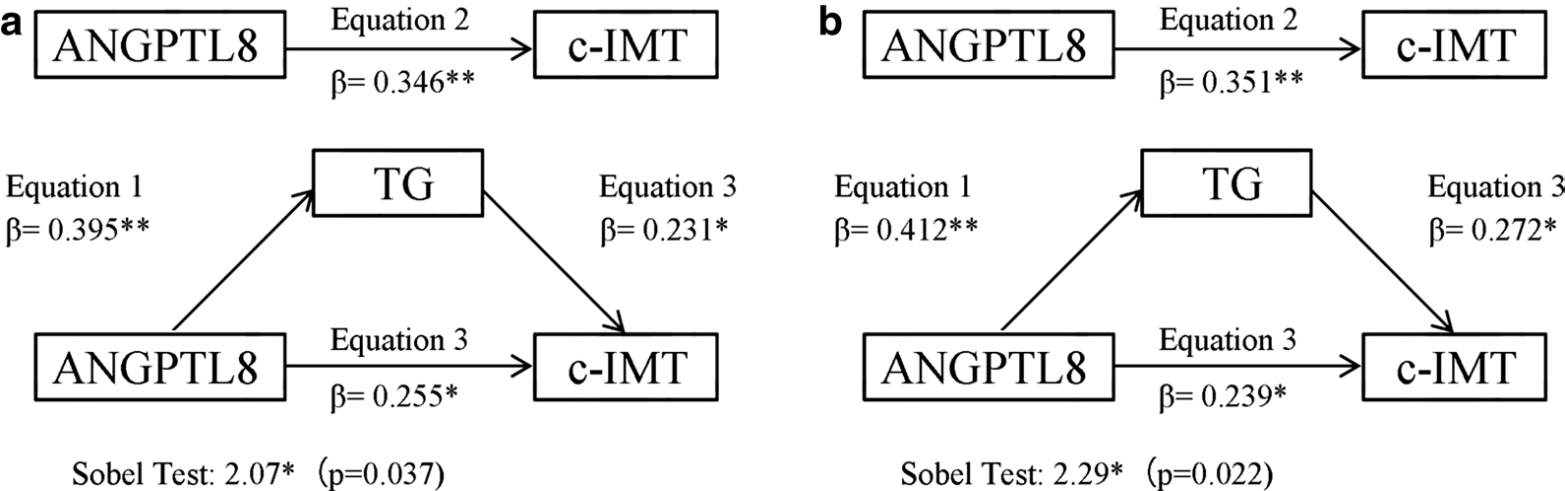
**a** The mediator role of TG in the relationship between ANGPTL8 and c-IMT in type 2 diabetic patients without subclinical atherosclerosis group. **b** The mediator role of TG in the relationship between ANGPTL8 and c-IMT in type 2 diabetic patients with subclinical atherosclerosis group

The mediation analysis revealed that TG partially mediated the positive relationship between ANGPTL8 and c-IMT in both type 2 diabetic patients with and without subclinical atherosclerosis groups (Fig. 2). When we tested the mediator role of TG in the relationship between ANGPTL8 and c-IMT in type 2 diabetic patients without subclinical atherosclerosis group (Fig. 2a), in the first linear regression equation, ANGPTL8 was positively associated with TG (P < 0.001). In the second equation, ANGPTL8 was positively associated with c-IMT (P < 0.001). In the third equation, when ANGPTL8 and TG were simultaneously included in the model, ANGPTL8 and TG were both positively associated with c-IMT (all P < 0.05). These data indicated that the effects of ANGPTL8 on c-IMT were partially mediated by TG. The Sobel Test for mediation estimated that the percentage of total effect (direct plus indirect) mediated by TG was 26.3% (z = 2.07; P = 0.037). The analysis of the mediator role of TG in the relationship between ANGPTL8 and c-IMT in type 2 diabetic patients with subclinical atherosclerosis group showed similar results (Fig. 2b), such that TG may be considered as partial mediator. The estimated percentage of total effect mediated by TG was 31.9% (z = 2.29; P = 0.022). Because fasting plasma glucose, HbA1c and HOMA-IR were not related to ANGPTL8 significantly, the mediator roles of hyperglycemia and insulin resistance in the relationship between ANGPTL8 and c-IMT were not further evaluated.

### Associations between ANGPTL8 and subclinical atherosclerosis in type 2 diabetes

Multivariate binary logistic regression revealed that serum ANGPTL8 was significantly related to subclinical atherosclerosis [odds ratio (OR) 3.05 (95% CI 1.59–5.86), P = 0.001] without adjustment. Following multiple adjustment with age, sex, and body mass index (BMI), we observed that the OR for TG was significantly related to subclinical atherosclerosis [1.30 (95% CI 1.04–1.63), P = 0.023], while ANGPTL8 had the highest OR and was significantly related to subclinical atherosclerosis [OR 2.90 (95% CI 1.48–5.70), P = 0.002] (Table 4).

**Table 4:** OR (95% CI) by binary logistic regression models for atherosclerosis

Models	Covariates	T2DM without atherosclerosis		T2DM with atherosclerosis	
		OR (95% CI)	P	OR (95% CI)	P
Model 1	Age	1.0	–	1.02 (0.98, 1.05)	0.371
	TG	1.0	–	1.34 (1.08, 1.67)	0.009
	Duration of diabetes	1.0	–	1.07 (0.99, 1.15)	0.082
	ANGPTL8	1.0	–	3.05 (1.59, 5.86)	0.001
Model 2	Age	1.0	–	–	
	TG	1.0	–	1.33 (1.06, 1.67)	0.013
	Duration of diabetes	1.0	–	1.07 (0.99, 1.15)	0.102
	ANGPTL8	1.0	–	3.04 (1.56, 5.94)	0.001
Model 3	Age	1.0	–	1.02 (0.98, 1.05)	0.398
	TG	1.0	–	1.33 (1.07, 1.66)	0.012
	Duration of diabetes	1.0	–	1.07 (0.99, 1.15)	0.085
	ANGPTL8	1.0	–	2.99 (1.56, 5.75)	0.001
Model 4	Age	1.0	–	1.01 (0.98, 1.05)	0.490
	TG	1.0	–	1.32 (1.05, 1.65)	0.016
	Duration of diabetes	1.0	–	1.07 (0.99, 1.16)	0.071
	ANGPTL8	1.0	–	2.92 (1.51, 5.65)	0.001
Model 5	Age	1.0	–	–	
	TG	1.0	–	1.30 (1.04, 1.63)	0.023
	Duration of diabetes	1.0	–	1.07 (0.99, 1.15)	0.087
	ANGPTL8	1.0	–	2.90 (1.48, 5.70)	0.002

Model 1 unadjusted, Model 2 age adjusted, Model 3 sex adjusted, Model 4 BMI adjusted, Model 5 age, sex, and BMI adjusted

## Discussion

To our knowledge, the current study is the first to explore the potential mediating effect of hypertriglyceridemia, insulin resistance and hyperglycemia on the relationship between serum levels of ANGPTL8 and c-IMT and the possibility of identifying ANGPTL8 as a risk biomarker for subclinical atherosclerosis in type 2 diabetes through cross-sectional study in China. Four main results of the current study should be emphasized. First, serum levels of ANGPTL8 were increased in type 2 diabetic patients with subclinical atherosclerosis than in type 2 diabetic patients without subclinical atherosclerosis and control subjects. Second, increased serum levels of ANGPTL8 were positively associated with c-IMT in type 2 diabetes. Third, TG partially mediated this positive relationship between ANGPTL8 and c-IMT, whereas insulin resistance and hyperglycemia did not play a mediating role in this relationship. Fourth, ANGPTL8 might serve as a novel risk biomarker for subclinical atherosclerosis in type 2 diabetes.

There have been a number of studies showing increased circulating levels of ANGPTL8 in type 2 diabetic patients [3, 5, 12, 13] and also studies on unaltered or decreased circulating levels of ANGPTL8 [14, 15]. The reason for this discrepancy may be due to the difference in race, sample size, study design, medicine status, use of ELISA kits and handling of blood samples. In the current study, our data in agreement with most previous reports that serum levels of ANGPTL8 were increased in type 2 diabetic patients as compared with control subjects. In addition, we further found that serum levels of ANGPTL8 were significantly higher in type 2 diabetic patients with subclinical atherosclerosis group than that in type 2 diabetic patients without subclinical atherosclerosis group. Moreover, a positive association was found between ANGPTL8 and c-IMT in both type 2 diabetic patients with and without subclinical atherosclerosis groups. Although the causal relationship between ANGPTL8 and c-IMT can not be determined in this cross-sectional study, we still tried to investigate this relationship by asking what factors mediate this positive relationship between ANGPTL8 and c-IMT.

The relationship between ANGPTL8 and TG has well been established in animal models, mice lacking ANGPTL8 have significantly lower levels of TG than control mice [4], whereas overexpression of ANGPTL8 leads to increased levels of TG [16]. In agreement with these basic findings, our current data and other epidemiological evidence in humans indicated that serum levels of ANGPTL8 were positively and independently correlated with TG [17–19]. However, some other studies failed to find this significant correlation in type 2 diabetes [12, 20], the reason for this inconsistency might be partly attributed to the fact that many of the participants could have been treated with TG-lowering drugs which would affect TG levels, in our study, we excluded participants with any use of possible or known drugs affecting TG for more than 3 months or at any time within 12 months before the recruitment. In addition, mounting evidence suggested that TG may stimulate atherogenesis through production of proinflammatory cytokines, fibrinogen and coagulation factors and impairment of fibrinolysis [21, 22]. Consistently, our data showed a positive correlation between TG and c-IMT. Our mediation analysis revealed that as ANGPTL8 increased, c-IMT increased, but when the influence of TG was controlled, the relationship between ANGPTL8 and c-IMT was mitigated, consequently, the influence of ANGPTL8 on c-IMT was partially mediated by TG. So one possible explanation for the relationship between ANGPTL8 and c-IMT is that ANGPTL8 is involved in the pathogenesis of atherosclerosis through regulating TG levels. Some evidence suggested that ANGPTL8 had also been linked to cholesterol metabolism [20], however, in this study, we did not find any significant association between ANGPTL8 and TC, HDL-C or LDL-C, this inconsistency might be partly attributed to the statin use in our study.

Aside from TG, ANGPTL8 had also been shown to be connected with blood glucose and insulin resistance [5, 12]. However, when we evaluated the possible relationship between ANGPTL8 and blood glucose or insulin resistance, no significant relationship was found between blood glucose, insulin resistance and ANGPTL8 in type 2 diabetes, this result corresponded with the observation by Fenzl et al. [20]. Notably, the significant relationship between blood glucose, insulin resistance and ANGPTL8 was mostly found in newly diagnosed type 2 diabetes [5, 12], whereas the nonsignificant relationship was mostly found in treated type 2 diabetes [20]. Consequently, it is speculated that antidiabetic treatments might, at least in part, lead to this obscure or nonsignificant relationship between blood glucose, insulin resistance and ANGPTL8 in type 2 diabetes. The mediator effects of fasting plasma glucose, HbA1c and HOMA-IR on the relationship between ANGPTL8 and c-IMT were not further evaluated because of this nonsignificant association.

Recent studies have confirmed a link between circulating levels of ANGPTL8 and cardiovascular disease related biomarkers such as metabolic syndrome and inflammatory cytokines [23–26]. In three studies [23, 25, 26], for example, Abu-Farha et al., Crujeiras et al. and Liu et al. all found that circulating levels of ANGPTL8 were higher in participants with metabolic syndrome than those without, participants with higher ANGPTL8 levels experienced higher odds of having metabolic syndrome [23, 26]. With regard to the relationship between ANGPTL8 and inflammatory cytokines, Abu-Farha et al. also found a positive correlation between ANGPTL8 and HsCRP, suggesting that the inflammation might be connected to the increase in ANGPTL8 in humans that could result in aggravated cardiovascular disease. However, in the current study, the relationships between metabolic syndrome, inflammatory cytokines and ANGPTL8 were not assessed.

Binary logistic regression analysis was used to estimate the association of ANGPTL8 with the odds of subclinical atherosclerosis in type 2 diabetes, it revealed that elevated levels of ANGPTL8 were significantly associated with an increased risk of subclinical atherosclerosis in type 2 diabetes. Together our data may point towards a novel role for ANGPTL8 in the pathogenesis of atherosclerosis in type 2 diabetic patients, whereas in control subjects with normal glucose tolerance the potential deleterious effects of ANGPTL8 could still be compensated.

Despite the significant results of our current study, several limitations should be considered. First, this study is an epidemiological cross-sectional research and somehow fails to address the causal role of ANGPTL8 in the pathogenesis of subclinical atherosclerosis in type 2 diabetes, which is needed to be elucidated by further investigation. Second, the positive relationship between ANGPTL8 and c-IMT is probably related to more than a single mediator variable (TG), further research is still needed to clarify more potential mediator role of other unknown factors. Third, this study fails to investigate the relationships between ANGPTL8 and cardiovascular disease related biomarkers such as metabolic syndrome and inflammatory cytokines. Fourth, ANGPTL8 levels were not further quantified using different human ANGPTL8 kits to confirm the results of this study. Fifth, generalizations based on our results could be limited because of unmeasured confounders.

## Conclusions

In summary, results of the current study revealed that serum levels of ANGPTL8 were significantly increased in type 2 diabetic patients with subclinical atherosclerosis, moreover, our data provided the first evidence that ANGPTL8 was positively correlated with c-IMT in this population and TG partially mediated this positive correlation, suggesting ANGPTL8 to be a new biomarker for subclinical atherosclerosis in type 2 diabetes. Further studies are needed to validate the causative relationship between ANGPTL8 and subclinical atherosclerosis in type 2 diabetes.

## Abbreviations

ANGPTL8: angiopoietin-like protein 8
BMI: body mass index
c-IMT: common carotid artery Intima-Media Thickness
FPG: fasting plasma glucose
HOMA-IR: homeostasis model assessment of insulin resistance
HDL-C: high-density lipoprotein-cholesterol
LDL-C: low-density lipoprotein-cholesterol
NGT: normal glucose tolerance
OR: odds ratio
RIFL: refeeding-induced fat and liver
TG: triglyceride
TC: total cholesterol
